# Single-Cell Measurements of Fixation and Intercellular Exchange of C and N in the Filaments of the Heterocyst-Forming Cyanobacterium *Anabaena* sp. Strain PCC 7120

**DOI:** 10.1128/mBio.01314-21

**Published:** 2021-08-17

**Authors:** Mercedes Nieves-Morión, Enrique Flores, Martin J. Whitehouse, Aurélien Thomen, Rachel A. Foster

**Affiliations:** a Department of Ecology, Environment and Plant Sciences, Stockholm University, Stockholm, Sweden; b Instituto de Bioquímica Vegetal y Fotosíntesis, CSIC and Universidad de Sevilla, Seville, Spain; c Department of Geosciences, Swedish Museum of Natural History, Stockholm, Sweden; d Department of Chemistry and Molecular Biology, University of Gothenburg, Göteborg, Sweden; Oregon State University

**Keywords:** LG-SIMS, NanoSIMS, carbon fixation, cyanobacteria, intercellular communication, nitrogen fixation

## Abstract

Under diazotrophic conditions, the model filamentous, heterocyst-forming cyanobacterium *Anabaena* sp. strain PCC 7120 develops a metabolic strategy based on the physical separation of the processes of oxygenic photosynthesis, in vegetative cells, and N_2_ fixation, in heterocysts. This strategy requires the exchange of carbon and nitrogen metabolites and their distribution along the filaments, which takes place through molecular diffusion via septal junctions involving FraCD proteins. Here, *Anabaena* was incubated in a time course (up to 20 h) with [^13^C]bicarbonate and ^15^N_2_ and analyzed by secondary ion mass spectrometry imaging (SIMS) (large-geometry SIMS [LG-SIMS] and NanoSIMS) to quantify C and N assimilation and distribution in the filaments. The ^13^C/^12^C and ^15^N/^14^N ratios measured in wild-type filaments showed a general increase with time. The enrichment was relatively homogeneous in vegetative cells along individual filaments, while it was reduced in heterocysts. Heterocysts, however, accumulated recently fixed N at their poles, in which the cyanophycin plug [multi-l-arginyl-poly(l-aspartic acid)] is located. In contrast to the rather homogeneous label found along stretches of vegetative cells, ^13^C/^12^C and ^15^N/^14^N ratios were significantly different between filaments both at the same and different time points, showing high variability in metabolic states. A *fraC fraD* mutant did not fix N_2_, and the ^13^C/^12^C ratio was homogeneous along the filament, including the heterocyst in contrast to the wild type. Our results show the consumption of reduced C in the heterocysts associated with the fixation and export of fixed N and present an unpredicted heterogeneity of cellular metabolic activity in different filaments of an *Anabaena* culture under controlled conditions.

## INTRODUCTION

Heterocyst-forming cyanobacteria of the order *Nostocales* grow as long chains of cells (filaments or trichomes), in which the absence of combined nitrogen (diazotrophic conditions) triggers a cell differentiation process resulting in two cell types along filaments: vegetative cells and heterocysts (1). Whereas expression of the CO_2_-fixing enzyme ribulose-1,5-bisphosphate carboxylase/oxygenase (Rubisco) takes place in the vegetative cells (2, 3), expression of the N_2_-fixing enzyme nitrogenase takes place in the heterocysts (3–6). N_2_ fixation is the process of reducing dinitrogen to ammonium. Heterocyst differentiation involves a specific program of gene expression producing filaments that contain intercalary heterocysts that, in most cases, are spaced by 10 to 15 vegetative cells along the filament (1). This process is a way to separate oxygenic photosynthesis and N_2_ fixation, which are incompatible metabolic activities because nitrogenase is inactivated in the presence of oxygen (7). Because of the segregation of CO_2_ fixation and N_2_ fixation in different cell types, an intercellular transfer of nutrients is needed in the filament, in which carbon (C)- and nitrogen (N)-containing metabolites are exchanged between cells allowing diazotrophic growth (1).

The intercellular molecular exchange in filamentous cyanobacteria has been studied in real time by fluorescence recovery after photobleaching (FRAP) analysis using fluorescent markers (8), which has indicated that exchange takes place by means of molecular diffusion through cell-cell joining structures termed septal junctions (9; reviewed in references 10 and 11). Most studies have been performed with *Anabaena* sp. strain PCC 7120 (hereafter *Anabaena*) that is a model for *Nostocales*. FRAP analysis carried out with several mutants of *Anabaena* has shown that proteins such as SepJ, FraC, and FraD are required for proper intercellular molecular exchange (8, 12, 13). FraC and FraD are involved in the septal junctions that have been recently visualized by cryoelectron tomography (14). *fraC* and/or *fraD* mutants produce short filaments with terminal heterocysts that show significantly low nitrogenase activity measured by acetylene reduction assays (12, 15).

Different studies using radioactive and stable isotopically labeled substrates ([^14^C]bicarbonate, [^13^C]bicarbonate, ^13^N_2_, and ^15^N_2_) have attempted to visualize and quantify CO_2_ and N_2_ fixation in a few heterocyst-forming cyanobacterial strains (2, 16–18). These studies showed that CO_2_ and N_2_ fixation takes place in vegetative cells and heterocysts, respectively, and that the fixed products are subsequently transported rapidly (2, 16). Moreover, heterocysts contain less recently fixed ^13^C and ^15^N than vegetative cells (17, 19), and there is substantial variation in the cellular and subcellular distribution of C and N, with heterocysts showing lower C/N ratios than vegetative cells (18). A study specifically comparing C and N enrichment and distribution between different filaments in a culture is however not available.

By providing the model cyanobacterium *Anabaena* with ^15^N-labeled N_2_ and ^13^C-labeled NaHCO_3_, here we performed stable isotope labeling experiments coupled to secondary ion mass spectrometry (SIMS). Two CAMECA instruments, a large-geometry (LG)-SIMS IMS 1280 and a NanoSIMS 50L, were used to image, measure, and describe the pattern of C and N_2_ fixation and the distribution of labeled C- and N-containing compounds at the cellular level along filaments. In parallel, we made the same isotope labeling incubations and measurements along the filaments in an *Anabaena* Δ*fraC* Δ*fraD* double mutant. Using the combined platforms enabled a high throughput of measurements on numerous filaments (about one hundred for each strain) across several time points, combined with fewer but higher-resolution measurements along the filaments. Our results provide insights into different patterns of C and N distribution in cyanobacterial filaments that result from variability in metabolic states despite highly controlled culture conditions.

## RESULTS

### C and N fixation and distribution along filaments.

**FIG 1:**
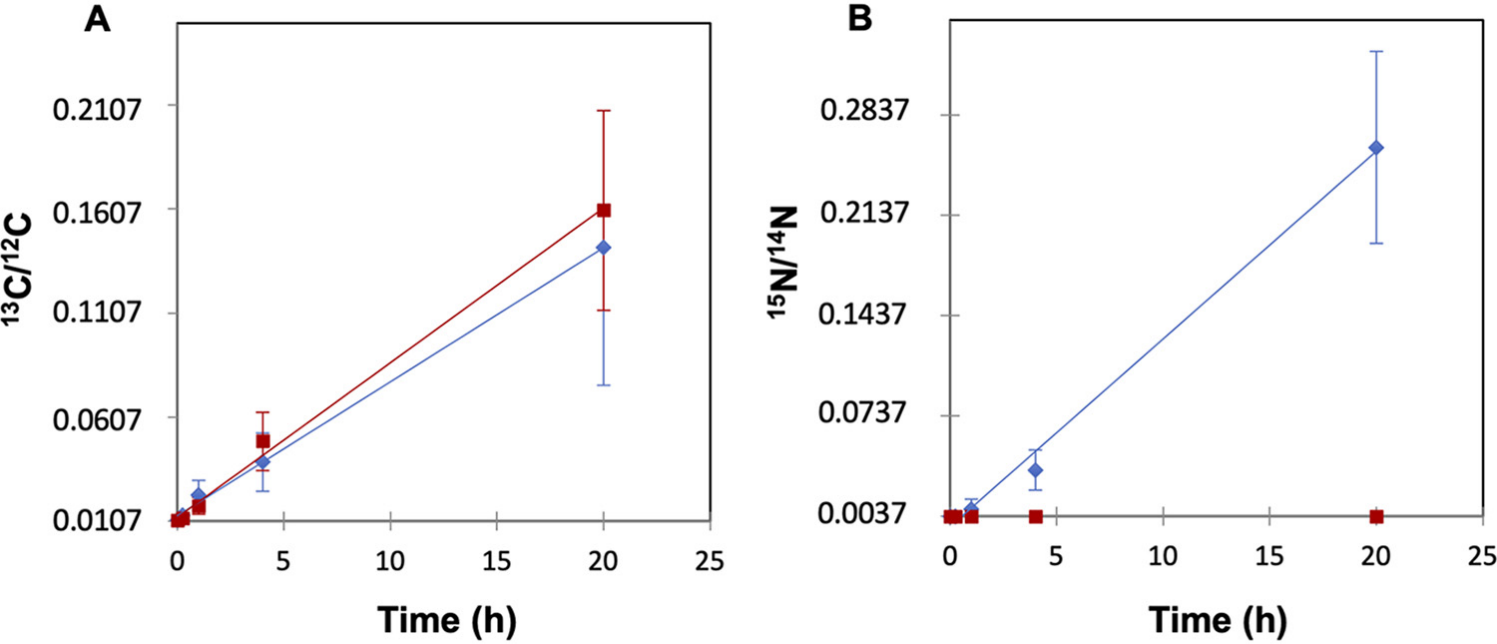
^13^C/^12^C ratios (A) and ^15^N/^14^N ratios (B) of cultures of *Anabaena* sp. strain PCC 7120 (WT) (blue diamonds) and strain CSVT22 (Δ*fraC* Δ*fraD*) (red squares). Samples were collected after 0, 15 min, 1 h, 4 h, and 20 h of incubation with ^13^C-labeled bicarbonate and ^15^N_2_. Previously, the cells had been induced for 48 h in the absence of combined N. The minimum values in the *y* axes correspond to the ^13^C/^12^C and ^15^N/^14^N ratio natural abundances, 0.0107 and 0.0037, respectively. The depicted data are the means ± standard deviations (SD) (error bars) of the LG-SIMS data collected from all cells analyzed for the different time points (WT, *n* = 259 to 427; CSVT22, *n* = 60 to 405) adjusted by linear regression.

The enrichment in ^13^C and ^15^N, resulting from incubation with ^13^C-labeled NaHCO_3_ and ^15^N-labeled N_2_ was measured and visualized as ^13^C/^12^C and ^15^N/^14^N ratios using large-geometry secondary ion mass spectrometry (LG-SIMS) along 100 filaments of *Anabaena* wild type (WT) and 81 filaments of mutant strain CSVT22 (Δ*fraC* Δ*fraD*). The filaments were induced for heterocyst differentiation by incubation in medium lacking combined N for 48 h and then amended with the stable isotopes and incubated under culture conditions for 15 min, 1 h, 4 h, and 20 h. The ^13^C enrichment in both strains resulted in a similar pattern of increasing ^13^C/^12^C ratio with time (Fig. 1A). ^15^N enrichment (^15^N/^14^N) was also observed in the WT, whereas the ^15^N/^14^N ratio in the *fraC fraD* mutant was not greater than natural abundance (0.0037) at any time point. Hence, in contrast to the WT, N_2_ fixation was negligible in the mutant (Fig. 1B).

**FIG 2:**
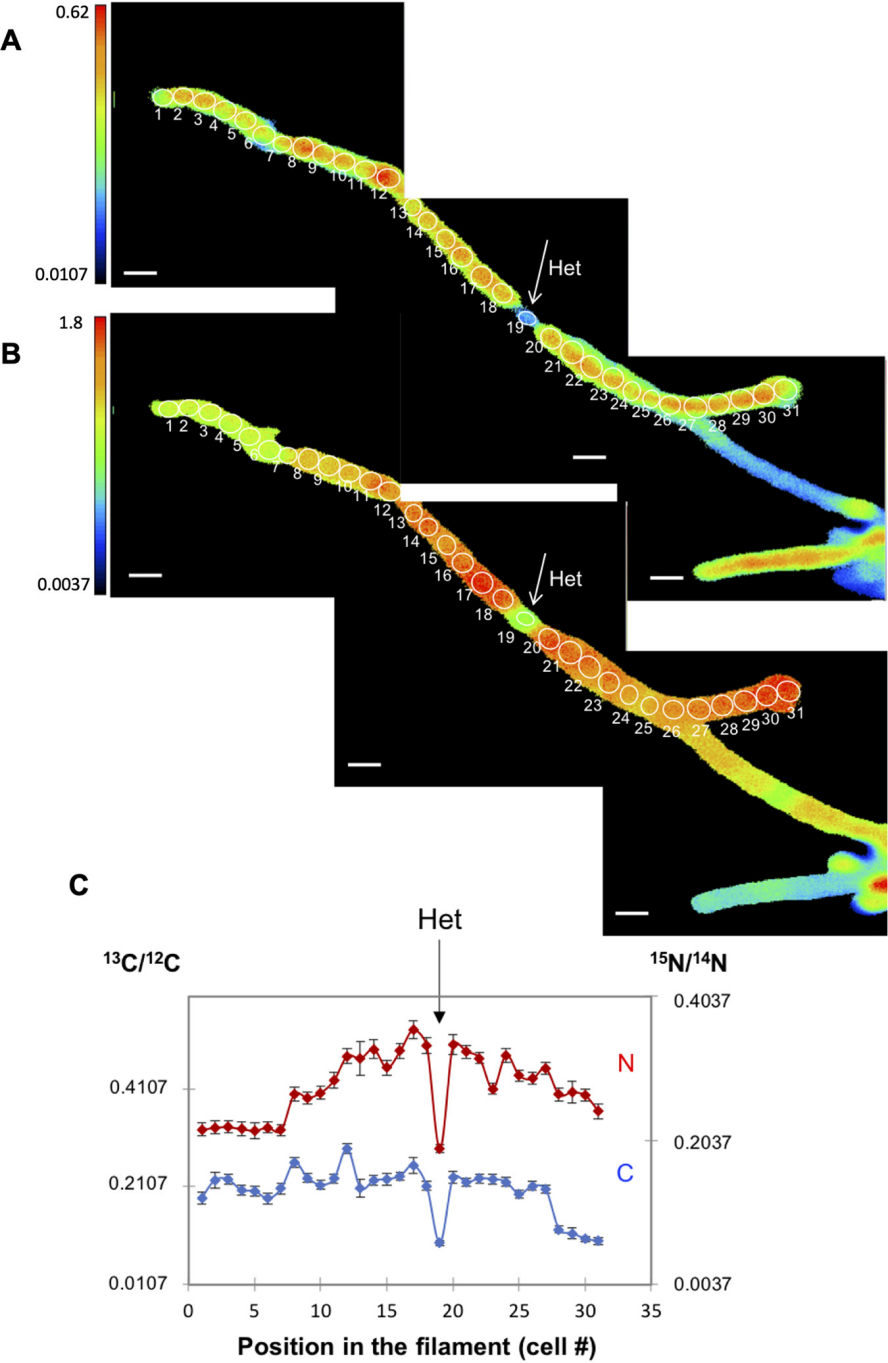
^13^C/^12^C and ^15^N/^14^N ratios in the cells along an individual filament of *Anabaena* WT after 20 h of incubation with the isotopically labeled substrates. The filament had been induced under combined N deprivation for 48 h before the addition of the isotopes. (A and B) The ^13^C/^12^C (A) and ^15^N/^14^N (B) ratio images collected from LG-SIMS imaging are shown. Color codes indicate ^13^C/^12^C (A) and ^15^N/^14^N (B) ratios, and the threshold is ^13^C or ^15^N natural abundance as indicated on the *y* axes. The numbers and white outlines denote the regions of interest (ROIs) used to calculate ^13^C/^12^C and ^15^N/^14^N ratios, which are shown in panel C. In all LG-SIMS analysis, data are presented as mean ± SD of 60 planes for each ROI. The location of one heterocyst (Het) is indicated. Bars, 5 μm.

**FIG 3:**
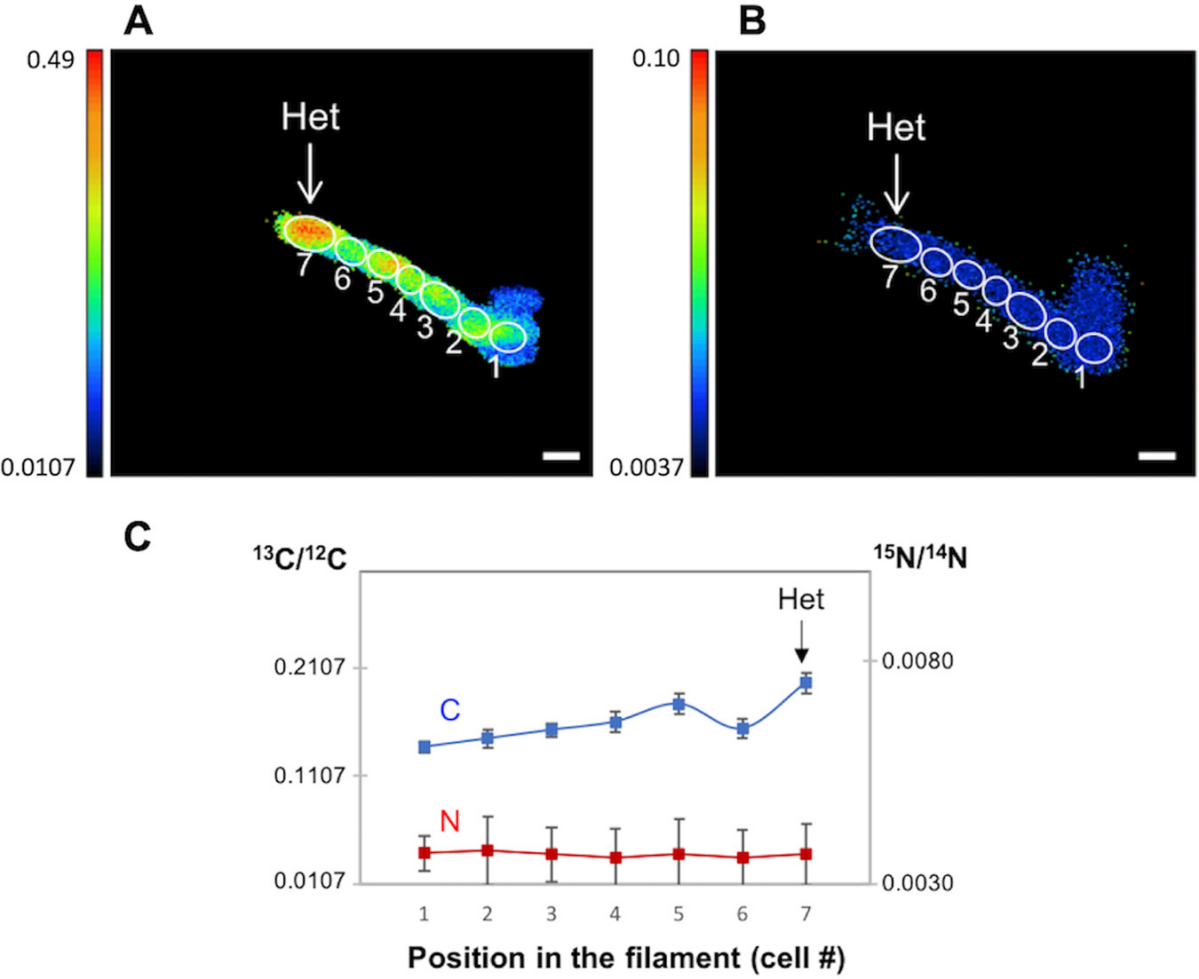
^13^C/^12^C and ^15^N/^14^N ratios in the cells along an individual filament of strain CSVT22 (Δ*fraC* Δ*fraD*) after 20 h of incubation with the isotopically labeled substrates. The filament had been induced under combined N deprivation for 48 h before the addition of the isotopes. (A and B) The ^13^C/^12^C (A) and ^15^N/^14^N (B) ratio images collected from LG-SIMS imaging are shown. Color codes indicate ^13^C/^12^C (A) or ^15^N/^14^N (B) ratios. The numbers and white outlines denote the regions of interest (ROIs) used to calculate ^13^C/^12^C and ^15^N/^14^N ratios (mean ± SD of 60 planes), which are shown in panel C. Note that the ^15^N/^14^N ratios correspond to the natural abundances of the isotopes. The location of one heterocyst (Het) is indicated. Bars, 5 μm.

In the preparation of samples for LG-SIMS, the fixation procedure affects cellular morphology, but heterocysts could be identified microscopically by their larger size compared to the vegetative cells (see Materials and Methods). Nine intercalary heterocysts were unequivocally identified in the WT and 20 terminal heterocysts in the *fraC fraD* mutant (note that analysis of the mutant was facilitated by its characteristic development of one terminal heterocyst in a short filament [15]). In the WT, the clearest example of a filament with an intercalary heterocyst was found after 20 h of incubation, in which ^13^C/^12^C and ^15^N/^14^N ratios were extremely high in the vegetative cells (^13^C/^12^C ratio, 0.0994 to 0.2881; ^15^N/^14^N ratio, 0.2176 to 0.3571) with an obvious decrease of both labels in the intercalary heterocyst (^13^C/^12^C ratio, 0.0952; ^15^N/^14^N, 0.1922) (Fig. 2). This observation likely reflects the transfer of fixed ^15^N from the heterocyst to the vegetative cells along the filament and the consumption of the ^13^C-labeled compounds received from the vegetative cells in the heterocyst. Interestingly, whereas the enrichment of ^13^C was quite homogeneous in most cells along the filament, the ^15^N enrichment decreased from the first or second vegetative cells in either direction along the filament from the heterocyst (Fig. 2C). In contrast, in the *fraC fraD* mutant, the ^13^C enrichment in a terminal heterocyst was similar to that in neighboring vegetative cells (Fig. 3; see also Fig. 10 below), likely reflecting lack of consumption of the ^13^C compounds received from the vegetative cells that, in the *fraC fraD* mutant, are not used for N_2_ fixation.

**FIG 4:**
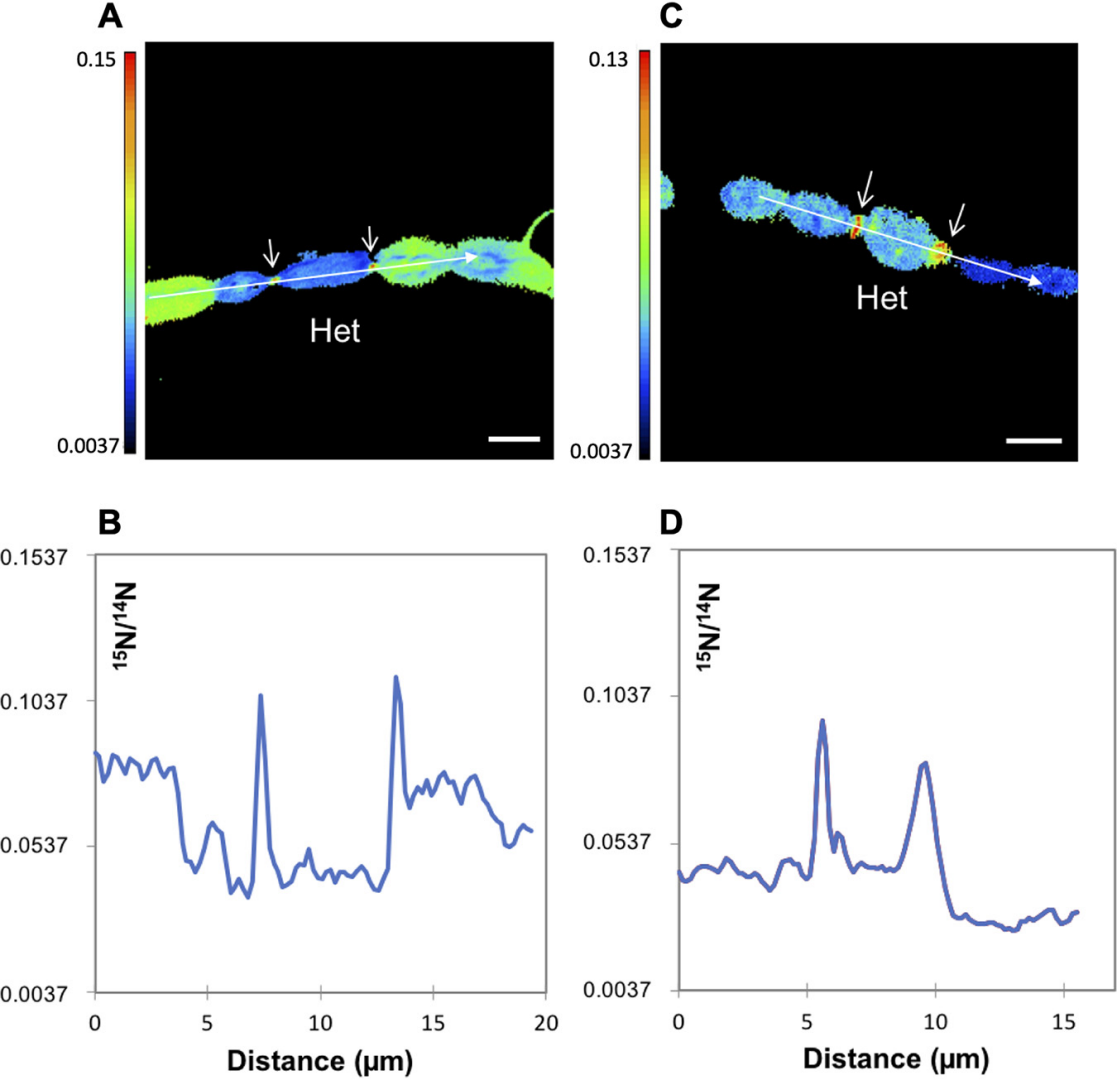
Two examples of ^15^N/^14^N ratio in *Anabaena* WT filaments collected by NanoSIMS imaging after 4 h of incubation with the isotopically labeled substrates. (A and C) ^15^N/^14^N ratio images from *Anabaena* filaments showing high ^15^N enrichment at the heterocyst necks (arrows). Color codes indicate ^15^N/^14^N ratios. (B and D) ^15^N/^14^N ratio along the lines indicated as longitudinal arrows in the images in panels A and C, respectively (data are the means from 33 and 3 planes, respectively). Het, heterocyst. Bars, 3 μm. ^13^C/^12^C ratios were in the range shown in Fig. 6C (4 h).

**FIG 5:**
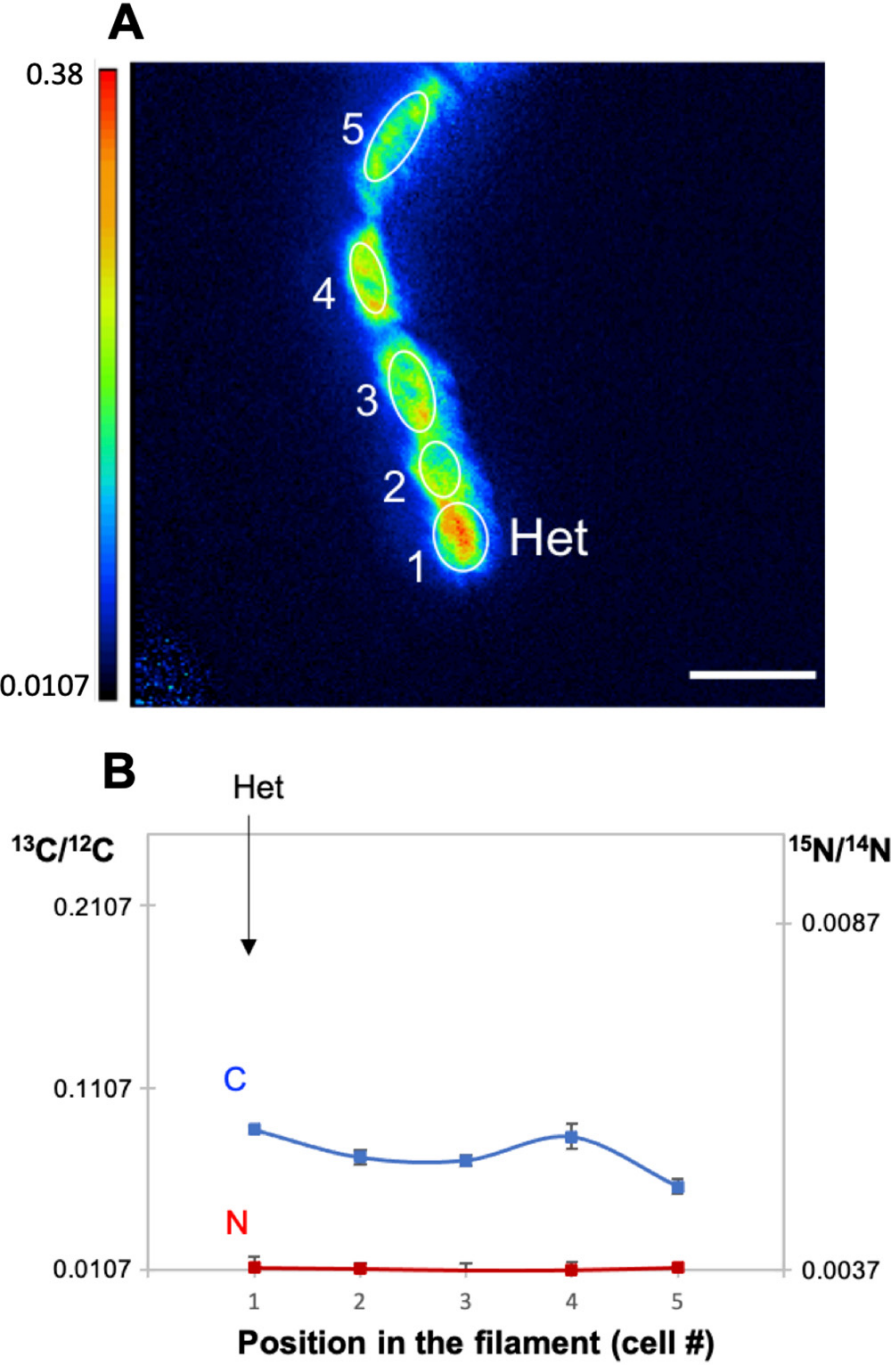
^13^C/^12^C and ^15^N/^14^N ratios in the cells along a filament of *Anabaena* mutant CSVT22 (Δ*fraC* Δ*fraD*) after 4 h of incubation with the isotopically labeled substrates. The ^13^C/^12^C ratio image collected from NanoSIMS imaging is shown in panel A. Color code indicates ^13^C/^12^C ratio. The numbers in the image denote regions of interest (ROIs) used to calculate ^13^C/^12^C and ^15^N/^14^N ratios (mean ± SD of four planes), which are shown in panel B. Het, heterocyst. Bar, 3 μm.

Subsequently, to investigate the ^13^C and ^15^N enrichment in *Anabaena* WT and *fraC fraD* mutant filaments with a higher spatial resolution, a series of measurements were performed by NanoSIMS on two *Anabaena* WT filaments (Fig. 4) and three *fraC fraD* mutant filaments (one example shown in Fig. 5) incubated 4 h with the isotopes. In one of the WT filaments, the ^15^N/^14^N ratio was higher in the neighboring vegetative cells than inside the heterocyst (Fig. 4A and B); in contrast, in the other filament analyzed, the ^15^N/^14^N ratio in the neighboring vegetative cells was similar or lower than in the heterocyst (Fig. 4C and D). Since the ^15^N enrichment (^15^N/^14^N) in vegetative cells of the latter filament was lower, i.e., 0.0424 compared to 0.0796 in the vegetative cells of the first filament, it is possible that the heterocyst in the second filament started to fix N_2_ more recently than the heterocyst in the first filament resulting in a lower accumulation of recently fixed N in the neighboring vegetative cells. In both filaments, however, the ^15^N/^14^N ratio showed obvious subcellular areas of ^15^N enrichment at the heterocyst poles, where cyanophycin granules are located (20, 21). The NanoSIMS analysis in the *fraC fraD* mutant after 4 h of incubation with the isotopes verified the ^13^C enrichment in a terminal heterocyst and a lack of ^15^N enrichment along the whole filament confirming again inactivity for N_2_ fixation (Fig. 5).

### Comparison of C and N enrichment and distribution between different filaments.

**FIG 6:**
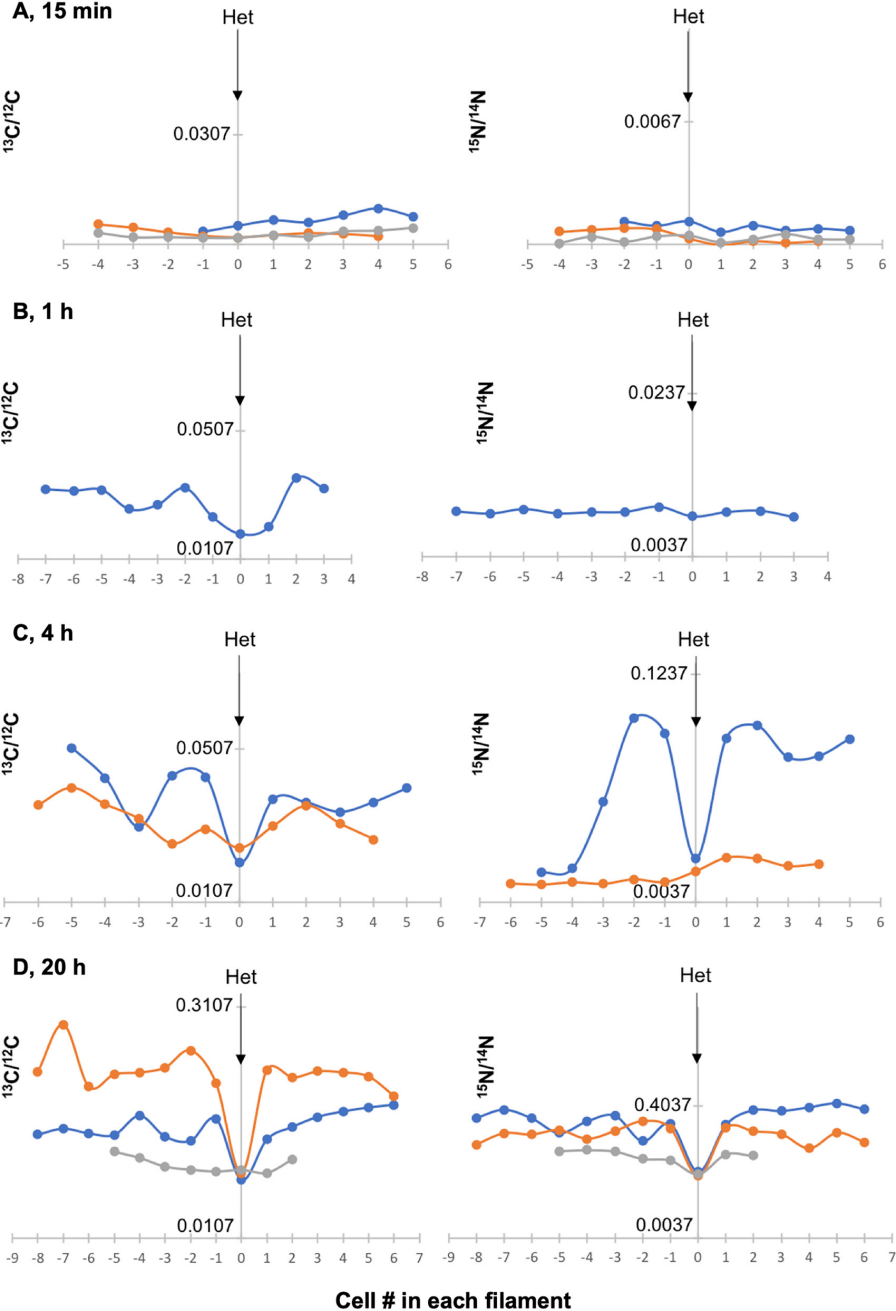
^13^C/^12^C and ^15^N/^14^N ratios in filaments of *Anabaena* WT collected by LG-SIMS after 15 min, 1 h, 4 h, and 20 h of incubation with the isotopes (one to three filaments for each time point). Heterocysts (Het) are indicated by arrows. Panel D includes in orange color the filament shown in Fig. 2. The *x* axis shows the numbering of the cells in the filaments taking the heterocyst as reference. The *y* axis shows the ^13^C/^12^C or ^15^N/^14^N ratio (note the different scales at the different time points).

To study further the variability in ^15^N enrichment, shown earlier for two heterocysts (belonging to two different filaments) and their adjacent vegetative cells (Fig. 4), we analyzed and compared C and N isotopic ratios at several time points (15 min, 1 h, 4 h, and 20 h) in the nine filaments in which heterocysts were identified (Fig. 6). At short incubation times (15 min and 1 h; Fig. 6A and B), the ^15^N enrichment could not be differentiated between heterocyst and vegetative cells, whereas the ^13^C enrichment was lower in the heterocyst than in the vegetative cells for a filament incubated for 1 h. When filaments from the 4-h and 20-h time points were analyzed (Fig. 6C and D), heterocysts from three filaments showed different enrichment ratios with respect to their adjacent cells, while the heterocysts and adjacent vegetative cells in two other filaments (one for each time point) were similarly low in both ^15^N/^14^N and ^13^C/^12^C ratios. In the heterocysts that had ^15^N/^14^N and ^13^C/^12^C ratios lower than the neighboring vegetative cells, gradients of enrichment of ^15^N as shown in Fig. 2 were not always observed. In summary, several heterocysts (observed at the 4-h and 20-h time points) showed lower ^15^N/^14^N and ^13^C/^12^C ratios than their adjacent vegetative cells, whereas the variation of isotope enrichment in vegetative cells appeared to be lower within filaments than between filaments. Nonetheless, a drastic change in enrichment can be occasionally observed along the vegetative cells within a filament, such as in the filament shown in blue in Fig. 6C (see ^15^N/^14^N ratios).

**FIG 7:**
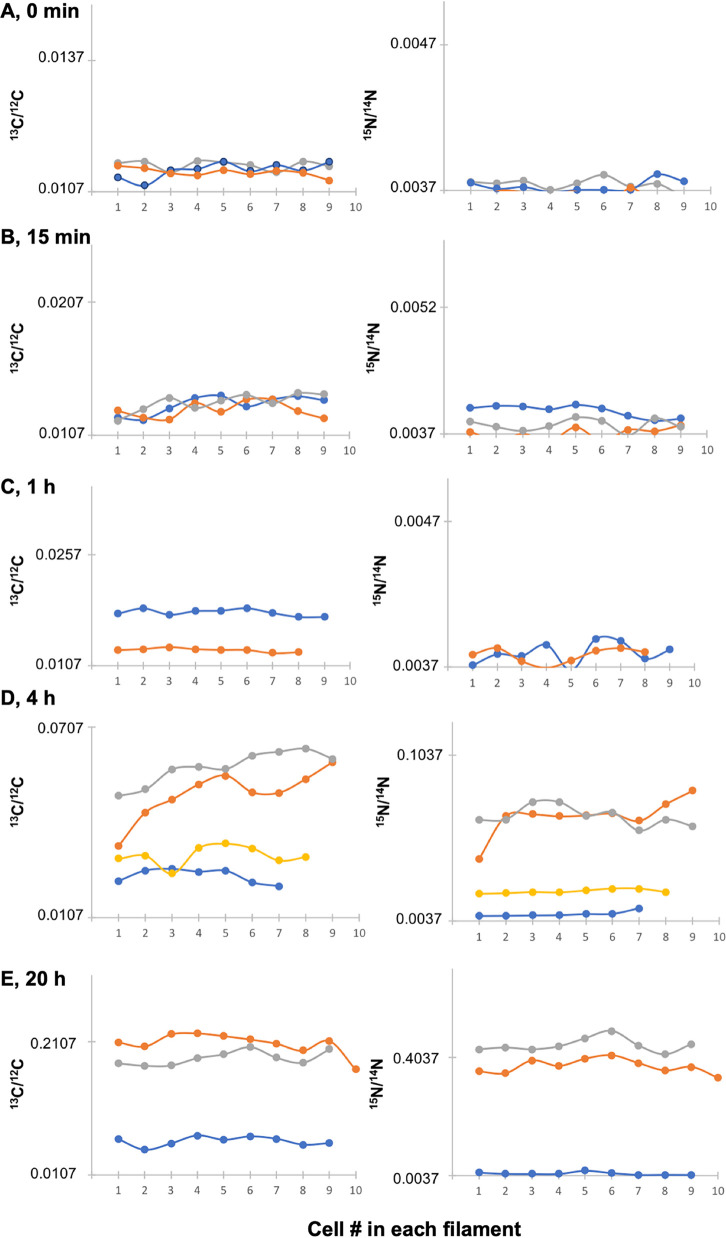
^13^C/^12^C and ^15^N/^14^N ratios in terminal stretches of filaments containing only vegetative cells of *Anabaena* WT collected by LG-SIMS at time zero and after 15 min, 1 h, 4 h, and 20 h of incubation with the isotopes (two to four filaments for each time point). Note the different scales for isotope ratios at the different time points. ^13^C/^12^C and ^15^N/^14^N ratios at 15 min were significantly different than at time zero (Student’s *t* test *P* < 0.001 for both isotopes).

**FIG 8:**
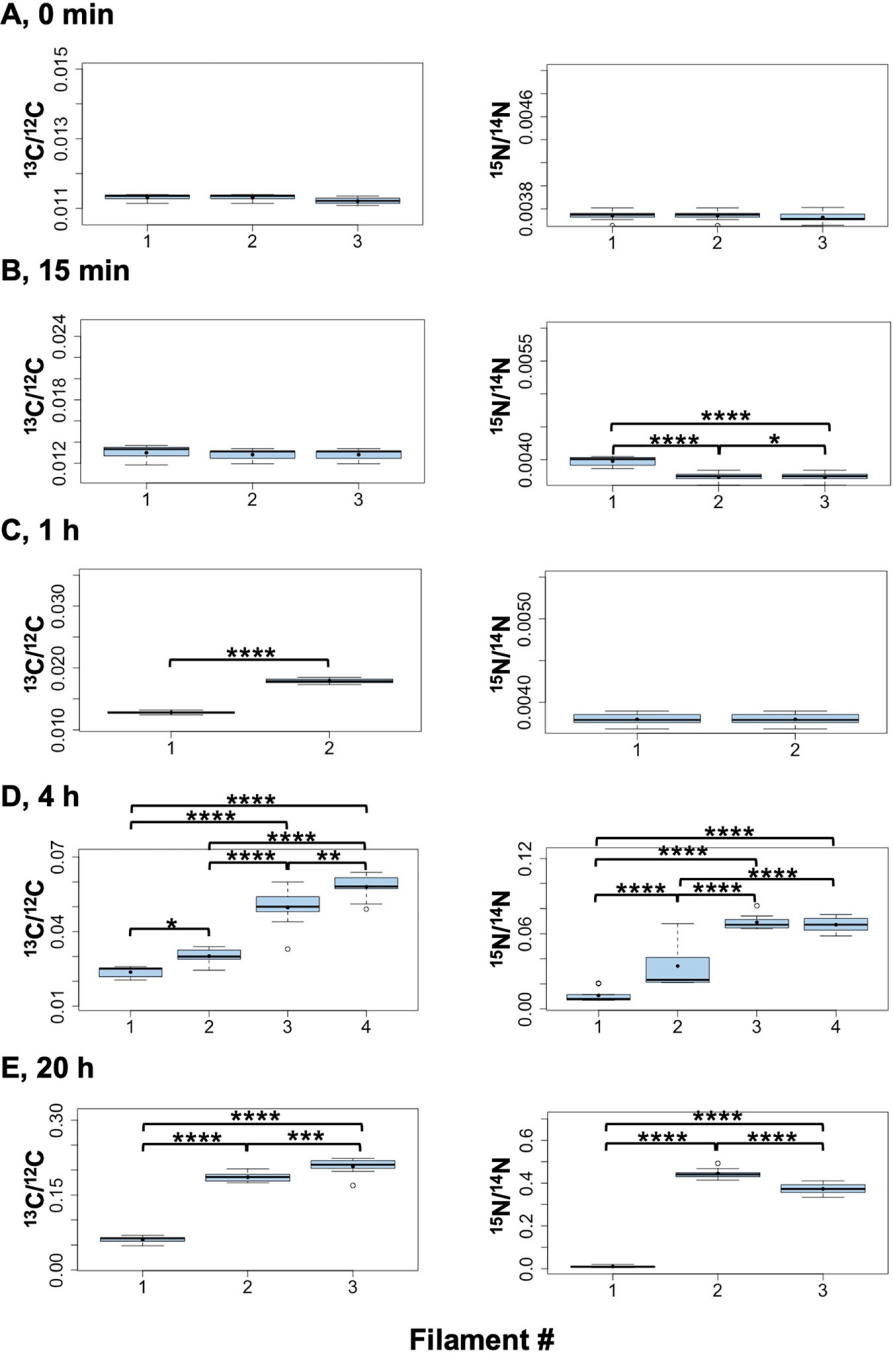
Box plot representation of the LG-SIMS data from Fig. 7. For each filament, data from all the cells were combined and presented in a box plot. Mean values are represented by black dots. On the *y* axes, the minimum values were the ^13^C and ^15^N natural abundances, 0.0107 and 0.0037, respectively. To assess the differences between filaments at each incubation time, the data were compared using the ANOVA test with Bonferroni correction (Bonferroni *post hoc* test). Statistical significance is indicated by asterisks as follows: *, 0.05 > *P* > 0.01; **, 0.01 > *P* > 0.001; ***, 0.001 > *P* > 0.0001; ****, *P* < 0.0001.

To compare further the enrichment pattern within and between individual WT filaments, a series of analyses were performed at all time points on terminal stretches of filaments containing only vegetative cells (unknown distance to/from heterocysts). Two to four filaments were measured for the different incubation times (Fig. 7). To facilitate statistical comparison of the different filaments at a given time of incubation, combined data for the cells in each filament were also analyzed (Fig. 8). As expected, the ^13^C/^12^C and ^15^N/^14^N ratios measured at time zero corresponded approximately to the natural abundances of the isotopes (0.0107 for ^13^C/^12^C and 0.0037 for ^15^N/^14^N; Fig. 7A and 8A). At 15 min, the ^13^C/^12^C and ^15^N/^14^N ratios started to increase but were not very different for the different cells (Fig. 7B), although these small differences were significant when comparing the ^15^N/^14^N ratios in different filaments (Fig. 8B). At 1 h, one of the two filaments analyzed showed a significantly higher ^13^C/^12^C enrichment, whereas the ^15^N/^14^N ratios were not significantly different (Fig. 7C and 8C). After 4 h of incubation, the ^13^C/^12^C and ^15^N/^14^N ratios were more similar for the cells within a filament than between filaments, although the cells in one filament showed substantial variability of ^13^C and ^15^N isotope enrichments (Fig. 7D; filament in orange color). Notably, the four filaments analyzed were significantly different from each other for both ^13^C/^12^C and ^15^N/^14^N ratios (with the only exception of filaments 3 and 4, in which the ^15^N/^14^N ratios were not different; Fig. 8D). A similar trend was observed after 20 h of incubation when again the ^13^C/^12^C and ^15^N/^14^N ratios were quite homogeneous for the cells along a filament (Fig. 7E). However, two of the three filaments analyzed showed high average ^13^C/^12^C and ^15^N/^14^N ratios, whereas the third filament was significantly reduced (Fig. 8E). Because a correlation between ^13^C and ^15^N enrichments was generally apparent for the different filaments (Fig. 7 and 8), we calculated the correlation coefficient between the mean ^13^C/^12^C and ^15^N/^14^N ratios for all the filaments analyzed from the different incubation times (15 min to 20 h) and found a value of 0.969, indicating a strong positive correlation between ^13^C and ^15^N enrichment. In summary, these analyses of stretches of vegetative cells in a number of different filaments from multiple time points showed that, in general, enrichments measured for both ^13^C and ^15^N correlated and were more homogenous along a single filament than between the cells of different filaments.

**FIG 9:**
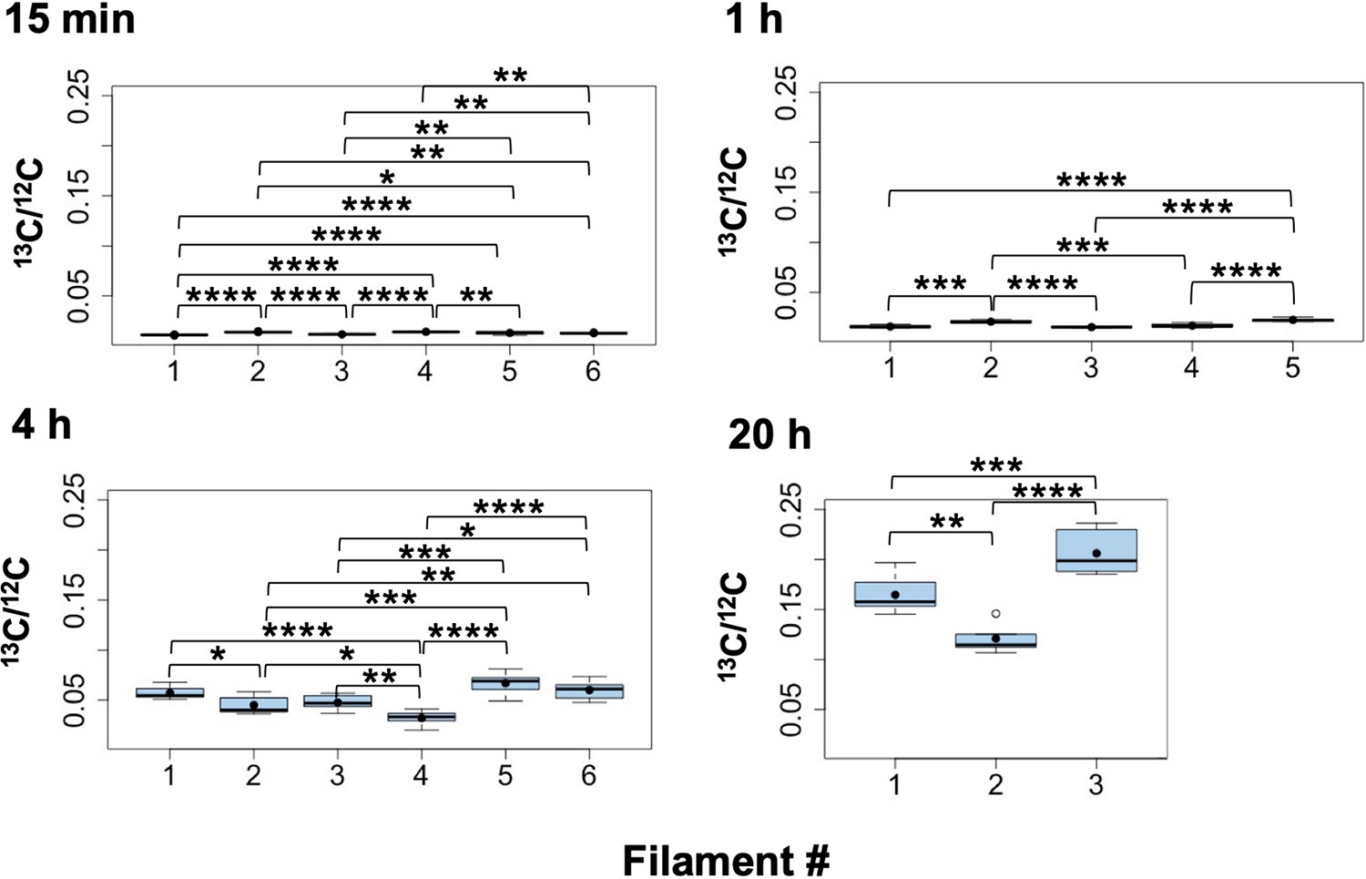
^13^C/^12^C ratios along individual filaments of *Anabaena* strain CSVT22 (Δ*fraC* Δ*fraD*) after 15 min, 1 h, 4 h, and 20 h of incubation with the isotopes. The filaments had been induced under combined N deprivation for 48 h. LG-SIMS data (^13^C/^12^C ratios, mean of 60 planes) collected from four to seven consecutive vegetative cells and one terminal heterocyst in each filament are depicted as box plots. The numbers of filaments analyzed were 6 at 15 min and 4 h, 5 at 1 h, and 3 at 20 h. The graph for the 20-h time point includes the filament shown in Fig. 3. On the *y* axes, the minimum values were the ^13^C natural abundance, 0.0107. Mean values are represented by black dots. To assess the differences between filaments at each incubation time, the data were compared using the ANOVA test with Bonferroni correction (Bonferroni *post hoc* test). Statistical significance: *, 0.05 > *P* > 0.01; **, 0.01 > *P* > 0.001; ***, 0.001 > *P* > 0.0001; ****, *P* < 0.0001.

**FIG 10:**
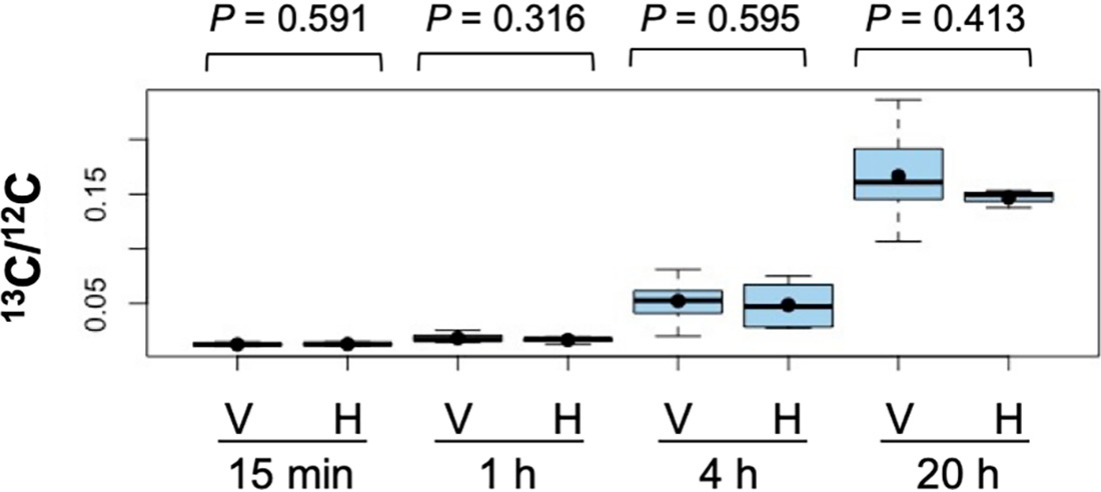
^13^C/^12^C ratios in vegetative cells and heterocysts of filaments of *Anabaena* strain CSVT22 (Δ*fraC* Δ*fraD*) after 15 min, 1 h, 4 h, and 20 h of incubation. LG-SIMS data (^13^C/^12^C ratios, mean of 60 planes) collected from 17 to 35 vegetative cells (V) and 3 to 6 heterocysts (H) for each time point are depicted as box plots. On the *y* axis, the minimum value was the ^13^C natural abundance, 0.0107. Student’s *t* test was performed to indicate the *P* values. Mean values are represented by black dots.

In the *fraC fraD* mutant (strain CSVT22), which grows as short filaments (15), statistical analyses of three to six filaments for each time point containing four to seven consecutive vegetative cells and one heterocyst in each filament showed a high homogeneity of ^13^C/^12^C ratios (low dispersion of the data) within filaments at each time point and over time (Fig. 9). Consistently, the cellular ^13^C/^12^C ratios in vegetative cells along filaments of the mutant in each time point showed no significant differences to the heterocysts at the corresponding time point (Fig. 10). On the other hand, different filaments showed significantly different ^13^C/^12^C ratios, exhibiting a variability in metabolic state between filaments as described above for the WT.

## DISCUSSION

The study of intercellular molecular exchange after C and N_2_ fixation in filamentous, heterocyst-forming cyanobacteria is crucial to understand the physiology and development of these organisms. Different methods have been used to study these processes in some cyanobacterial strains, including experimental studies using radioactive or stable isotopes and fluorescently labeled substrates (2, 8, 13, 16, 17). Stable isotopes and tracing by NanoSIMS have also been applied to a number of wild populations of heterocyst-forming cyanobacteria, including free-living and colonial strains (19, 22–24) as well as strains in symbiosis with diatoms (25). Here, we used the model heterocyst-forming cyanobacterium *Anabaena* to image the C and N_2_ fixation and cellular distribution of recently fixed C and N at several time points of incubation with stable isotopes (provided as [^13^C]bicarbonate and ^15^N_2_, respectively) coupled to LG-SIMS and NanoSIMS analyses. In this type of analysis, the cells that had been grown autotrophically with nutrients containing natural abundances of stable C isotopes (^13^C/^12^C, 0.0107) and stable N isotopes (^15^N/^14^N, 0.0037) will add ^13^C- and ^15^N-enriched material to the biomass characterized by those isotopic ratios at time zero. It is important to note that, in our experiments, a substantial part of the ^13^C- and ^15^N-enriched biomass will consist of macromolecules synthesized from ^13^C- and ^15^N-enriched metabolites produced in the fixation processes. Thus, the ^13^C/^12^C and ^15^N/^14^N ratios of the biomass increase with time (Fig. 1).

The transfer of newly fixed C from vegetative cells to heterocysts and of reduced N from heterocysts to vegetative cells are rapid processes that can be detected within minutes after amendments of radioactive isotopes (2, 16). In the case of N, there seems to be a gradient of recently fixed N that decreases away from the heterocyst (16). Similar to earlier work, enrichment with ^15^N in *Anabaena* was measurable within 4 to 20 h, and a decreasing gradient of recently fixed N could also be observed in some stretches of vegetative cells close to a heterocyst (Fig. 2), but this was not a general phenomenon (Fig. 6C and D). Nonetheless, we did observe variability in both ^13^C and ^15^N enrichment in the vegetative cells along filament stretches close to a heterocyst after different periods of C and N_2_ fixation (Fig. 6C and D). Communication between cells should contribute to homogenize the cells in a filament (8). However, recent studies have shown the presence in the filaments of *Anabaena* of noncommunicating cells, which are less abundant in diazotrophic cultures than in the presence of combined N (26). Noncommunicating or low-communicating cells could produce heterogeneous stretches of cells in a filament.

**FIG 11:**
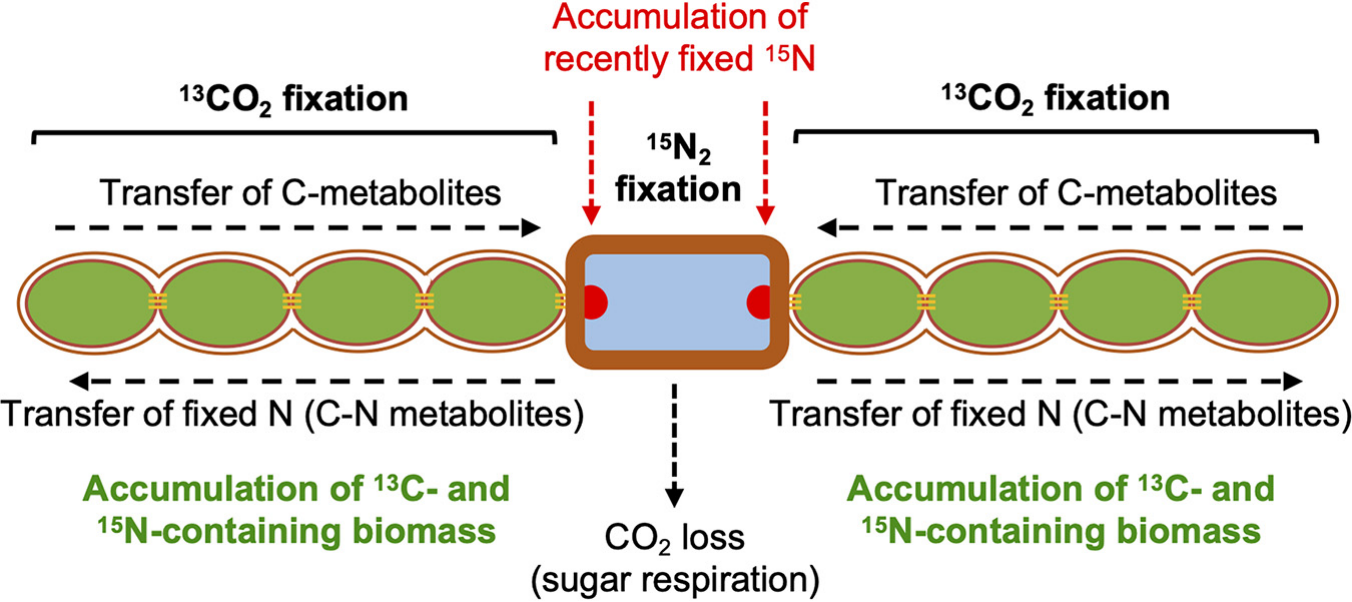
Scheme of carbon and nitrogen fixation and distribution in the diazotrophic filament of wild-type *Anabaena* (a filament consisting of a central heterocyst [note the thick envelope] and four vegetative cells at each side is depicted). As shown by incorporation of ^13^C and ^15^N from [^13^C]bicarbonate and ^15^N_2_, respectively, ^13^C- and ^15^N-labeled biomass accumulates mainly in the vegetative cells (see Fig. 2), although recently fixed N also accumulates at the cyanophycin granules (indicated as red semicircles) in the heterocyst poles (see Fig. 4). However, in the *Anabaena* Δ*fraC* Δ*fraD* mutant, which does not fix N_2_, ^13^C-labeled biomass also accumulates in the heterocysts (see Fig. 5). Therefore, in the wild type, ^13^C-labeled metabolites received from the vegetative cells that do not accumulate in the heterocysts are instead used (i) in respiration (releasing CO_2_) to fuel N_2_ fixation and (ii) for the incorporation of fixed N and transferred back to the vegetative cells as amino acids. The filament structure shown highlights the presence of septal junctions (thin horizontal orange lines) and of a continuous periplasmic space.

Within a filament, there can be a substantial difference in ^13^C and ^15^N enrichment between heterocysts and vegetative cells after 4 to 20 h of fixation of the isotopically labeled substrates, with both ^15^N/^14^N and ^13^C/^12^C ratios being lower in the heterocysts. This observation is consistent with previous data of NanoSIMS analysis in Anabaena oscillarioides, in which several time points during a diurnal cycle showed lower ^13^C and ^15^N enrichments in heterocysts than in vegetative cells (17). Additionally, some colony-forming N_2_-fixing cyanobacteria such as *Aphanizomenon* sp. and Nodularia spumigena also showed higher levels of ^13^C and ^15^N in vegetative cells than in heterocysts (19, 24). These observations likely reflect the fact that heterocysts are terminally differentiated cells that have stopped growing, and therefore, new C and N compounds are incorporated into cellular materials only at a low level (e.g., to support macromolecular turnover). Hence, most ^13^C compounds received from the vegetative cells are consumed in the heterocysts to fuel N_2_ fixation (e.g., sugars are respired down to CO_2_) and used for the incorporation of fixed N, and much of the recently fixed ^15^N is exported from the heterocysts to the vegetative cells (Fig. 11). In contrast, the heterocysts of the *fraC fraD* mutant, which shows no N_2_ fixation, had a level of ^13^C similar to that of the adjacent vegetative cells (Fig. 3, 5, and 10). Because the *fraC*, *fraD*, and *fraC fraD* mutants form well-developed heterocysts (12, 15), we consider it unlikely that the heterocysts of the *fraC fraD* mutant can fix CO_2_. Deletion of *fraC* and *fraD* impairs the intercellular transfer of fluorescent markers, including a sucrose analogue, esculin (13), and diazotrophic growth (15), suggesting that insufficient C supply could limit N_2_ fixation in the heterocysts. Our results show, however, that deletion of *fraC* and *fraD* does not prevent C transfer from vegetative cells to the terminal heterocyst since we observed a C metabolic balance along the filaments much more homogeneous than in WT filaments, likely triggered by no C consumption in the heterocyst to fuel N_2_ fixation. The inactive N_2_ fixation shown by the *fraC fraD* mutant might thus be related to a nonfunctional heterocyst rather than to C limitation. Alternatively, different types of septal junctions may transfer different compounds (13, 15), and the *fraC fraD* mutant could be impaired in the transfer of sucrose, which is essential for diazotrophic growth (27, 28), but not for instance of glutamate, which is needed for the assimilation of ammonia resulting from N_2_ fixation (29). Further work will be necessary to resolve this issue, but in any case, our results show a strong relationship between C consumption and N_2_ fixation in the heterocysts.

Imaging and measurements by NanoSIMS of subcellular regions quantified high ^15^N enrichment at the heterocyst necks. Many cyanobacteria are capable of converting fixed N into a storage compound known as cyanophycin (or cyanophycin granule polypeptide), which is an aspartate-arginine polymer [multi-l-arginyl-poly(l-aspartic acid)] that accumulates under unbalanced growth in the presence of sufficient N (30). In heterocyst-forming cyanobacteria, cyanophycin accumulates conspicuously at the heterocyst poles, where it is thought to be a key intermediate in the storage of fixed N (21). Thus, the high ^15^N-enrichment region at the heterocyst poles can correspond to cyanophycin granule polypeptide, and this observation suggests a strong accumulation of recently fixed N in the cyanophycin granules. This is consistent with a key role of a cyanophycin mobilization product, β-aspartyl-arginine dipeptide, in the transfer of fixed N from the heterocysts to the vegetative cells (31).

The variability in ^13^C and ^15^N enrichment between filaments (Fig. 7 and 8) might reflect different metabolic states of the filaments (or of filament stretches) during incubation with the isotopes. To recapitulate, filaments grown with combined N were incubated for 48 h in the absence of combined N to ensure the formation of N_2_-fixing heterocysts. In spite of synchronization in the first round of heterocyst differentiation (typically during the first 24 h after combined N removal [32]), it is possible that a second round of heterocyst differentiation took place during the 48-h incubation period before injecting the isotopes, and further differentiation could take place in the next hours of incubation. Thus, a heterogeneous population of filaments with heterocysts of different ages could constitute the culture, and in *Anabaena*, it has been shown that at 48 h of incubation without combined N, some heterocysts have lost communication (13). Consistently, filaments differentially labeled with fixed ^15^N (range of ^15^N/^14^N ratio in WT filaments, 0.0037 to 0.4927) were measured in our analysis, and filaments that showed negligible ^15^N enrichment also showed poor ^13^C enrichment. Because the cellular C/N balance is highly regulated in *Anabaena* (32), it is plausible that filaments of the WT that fixed little N were affected in CO_2_ fixation, as observed for unicellular cyanobacteria after several hours of N deprivation (33). On the other hand, different filaments could exhibit dissimilar metabolic states because they can be at different positions in the circadian cycle, which runs in individual *Anabaena* filaments even under constant light (34). Thus, the possibility that stretches of vegetative cells with low photosynthetic activity provide a heterocyst with insufficient reduced C affecting N_2_ fixation should also be considered. Indeed, the observation that filaments of the *fraC fraD* mutant (which does not fix N_2_) showed significant variability in ^13^C enrichment supports the idea that different filaments can be in different metabolic states. In contrast to the WT, the *fraC fraD* mutant fixed CO_2_ at high levels in spite of being deficient in N_2_ fixation. This might be related to an altered regulation of C assimilation as recently observed by transcriptomic analysis (35), which showed increased expression of genes encoding carbon uptake systems and some photosynthetic components in *Anabaena* mutants lacking septal proteins, including FraCD. In summary, our results have revealed the metabolic heterogeneity of individual cells and filaments in an *Anabaena* culture under controlled laboratory conditions, highlighting the interplay between C and N_2_ fixation and assimilation, which are highly regulated processes (32).

## MATERIALS AND METHODS

### Cyanobacterial strains and growth conditions.

*Anabaena* sp. (also known as *Nostoc* sp.) strains PCC 7120 (WT) and CSVT22 (Δ*fraC* Δ*fraD* mutant; 15) were grown in BG11 medium modified to contain ferric citrate instead of ferric ammonium citrate (36) at 30°C in continuous light (75 μmol m^−2^ s^−1^) in shaken (90 to 100 rpm) 50-ml liquid cultures. For the induction of heterocysts, the strains were incubated in BG11_0_ medium for 48 h after three washing steps with BG11_0_ medium (free of combined nitrogen). Chlorophyll *a* (Chl) content of cultures was determined by the method of Mackinney (37).

### ^15^N_2_ and [^13^C]bicarbonate incubations.

^15^N_2_ and [^13^C]bicarbonate incubations were performed with *Anabaena* WT and strain CSVT22. A time course experiment (0, 15 min, 1 h, 4 h, and 20 h) with cultures that had been induced for N_2_ fixation during 48 h, was performed prior to LG-SIMS and NanoSIMS analyses. Four replicate 175-ml bottles (one for each time point and strain) were filled for *Anabaena* and CSVT22 liquid cultures (BG11_0_ medium) at 1 μg Chl ml^−1^, capped and sealed without air bubbles, and subsequently amended through the septa cap with 10 ml of 99% ^15^N_2_ (Cambridge Isotope Laboratories Inc., Cambridge, MA, USA) and 1 ml of 87.5 mM NaH^13^CO_3_ (final concentration, 0.5 mM; about 72.5% labeling of ^13^C), except the bottle for time point 0, which was used as nonlabeled control. Given the short incubation period for the earlier time points (15 min to 4 h), we used a higher volume amendment of ^15^N_2_ gas than required for the desired final labeling percent (e.g., 10%) to account for the noninstantaneous dissolution of ^15^N_2_ (38). The bottles were incubated at the same light and temperature conditions used for the cyanobacterial growth and induction of heterocysts (described above). A subsample (8 ml) was collected at each time point (0, 15 min, 1 h, 4 h, and 20 h), fixed to a final concentration of 2% (wt/vol) with paraformaldehyde (PFA) for at least 1 h at room temperature and gently filtered by low vacuum (>5 lb/in^2^) onto a 3-μm-pore-size presputtered gold (Au) membrane filter (Millipore). After fixation, the filters were washed three times in 15 ml of BG11_0_ medium, and samples were stored dry at −20°C until further processing. One-milliliter samples for measuring the Chl content were taken at the end of the experiment.

### LG-SIMS and NanoSIMS analyses.

SIMS analysis was performed using a CAMECA IMS 1280 large-geometry (LG)-SIMS instrument, while NanoSIMS was performed on a CAMECA NanoSIMS 50L instrument. Sample preparation was similar for both. Briefly, a 5-mm sphere of the 25-mm presputtered gold filters containing the filaments was subsampled and fixed (using double-sided tape) onto a 25-mm-diameter covered glass slide, coated with a 5-nm-thick gold layer, prior to loading into the IMS 1280 microprobe instrument. For heterocyst identification in SIMS samples, an Olympus BX60 microscope and Olympus CellSens imaging system (NordSIM facility) were used after the LG-SIMS analyses. The heterocysts were distinguished morphologically based on a larger cell diameter and length; only heterocysts that were unequivocally identified were used for heterocyst-specific analyses. For NanoSIMS analyses, these 5-mm spheres were first observed on a laser microdissection microscope (LMD, Zeiss, CCI University of Gothenburg, Sweden) fitted with a triple filter (filter set 62 HE BFP GFP HcRed shift free) for fluorescence imaging; the emission bandwidths were 402 to 448 nm, 500 to 554 nm, and >615 nm for blue, green, and red fluorescence, respectively. The LMD was used to identify and make laser marks of filaments containing heterocysts so that these regions of interest could be quickly identified with the charge-coupled-device (CCD) camera of the NanoSIMS instrument. Fluorescence (Chl) and bright light images were also taken and used for orientation purposes during the SIMS analyses and postprocessing (see below). For each incubation time point, many different filaments of *Anabaena* WT and strain CSVT22 were analyzed (from 60 to 427 single-cell measurements for each strain) on the IMS 1280 microprobe instrument, while five *Anabaena* filaments and four CSVT22 filaments from the 15-min (data not shown here) and 4-h incubation times were imaged and measured by NanoSIMS.

For the LG-SIMS analyses, an initial 10 × 10 μm larger area than analyses (50 × 50 μm) was presputtered for 60 s using a ca. 3-nA Cs^+^ beam. The analysis region was then measured using a ca. 50-pA primary Cs^+^ beam combined with the dynamical transfer optical system in the secondary beam gated at 100% to facilitate high-mass resolution and high transmission from the rastered area. Following automated centering of the secondary beam in the field aperture and optimization of the mass calibration using the abundant ^12^C^14^N^−^ species, the CN signals were measured in peak switching mode in a single ion counting electron multiplier. Count times for the ^12^C^14^N^−^, ^13^C^14^N^−^, and ^12^C^15^N^−^ species were 1, 4, and 2 s, respectively, the full analysis comprising 60 cycles. A nominal mass (*M*) resolution of 11,900 (*M*/Δ*M*) was used in order to avoid interference on ^13^C^14^N^−^ from ^11^B^16^O.

Briefly, for the NanoSIMS, a similar approach was used; however, the following applies: cells were bombarded with a primary Cs^+^ beam and presputtered at 150 pA to deposit a dose of 10e^17^ Cs^+^ cm^−2^, followed by a rastering of the primary ion beam with a current between 2 pA and a beam size of 100 nm. The mass resolving power in all measurements was >10,000. The primary ion beam was used to raster the area of interest at a size of 15 to 30 μm^2^ with an image size of 256 × 256 pixels over the chosen raster size with a dwelling time of 5 ms per pixel. Negative charged secondary ions (^12^C^12^C^−^, ^12^C^13^C^−^, ^12^C^14^N^−^, and ^12^C^15^N^−^) were collected simultaneously in four parallel electron multiplier detectors of the multicollection system of the instrument. All scans (3 to 33 planes per filament) were corrected for drift of the beam and sample stage and accumulated after acquisition using the CAMECA WinImage2 software.

Isotope ratio images were created as the ratio of the sum of counts for each pixel over all recorded planes of the investigated isotope and the main isotope. Regions of interest (ROIs) were defined using the secondary electron (SE), ^12^C^14^N^−^, and epifluorescent images taken prior to the analysis. For each ROI, the isotopic ratio was calculated. WinImage2 was used on data from both LG- and NanoSIMS. SIMS statistical data analysis was performed based on box plot diagrams and the Student’s *t* test (confidence level, 95%) or analysis of variance (ANOVA) test using Bonferroni *post hoc* test (confidence level, 95%).

### Data availability.

The raw data of the different analyses described in this article will be made available upon request.
